# Identity and Temporal Fragmentation in Borderline Personality Disorder: A Systematic Review

**DOI:** 10.3390/brainsci14121221

**Published:** 2024-11-30

**Authors:** Ilaria Faggioli, Cecilia Maria Esposito, Giovanni Stanghellini

**Affiliations:** 1 Department of Health Sciences, University of Florence, 50121 Florence, Italy; ilaria.faggioli@edu.unifi.it (I.F.); ceciliam.esposito@hotmail.com (C.M.E.); 2 Department of Neurosciences and Mental Health, IRCCS Fondazione Ca’ Granda Ospedale Maggiore Policlinico, 20122 Milan, Italy; 3 Centro de Estudios de Fenomenologia y Psiquiatria, Diego Portales University, Santiago 8370067, Chile

**Keywords:** autobiographical memory, Borderline Personality Disorder, identity diffusion, lived time, narrative coherence, dynamic psychology

## Abstract

Background: Borderline Personality Disorder (BPD) is a prevalent psychopathological condition, affecting 0.7–2.7% of the general population. Given the known link between identity formation and the temporal, metacognitive, and narrative processes that contribute to its coherence, the aim of the present systematic review is to synthesize the current literature about the relationship between identity diffusion and lived time in adult patients with BPD. This would enhance knowledge and treatments, leading to a better understanding of the implications of time processes on identity diffusion in BPD. Methods: According to PRISMA guidelines, the main databases were consulted, and specific eligibility criteria were applied. The selection leads to the inclusion of 15 articles, investigating through integrated techniques the lived time, memory, self-reported narratives, and metacognition in BPD subjects. Results: A general agreement among researchers was found, confirming greater difficulty for BPD subjects in producing autobiographical stories, logically and temporally integrated, characterized by positive content. Functional and structural alterations were detected to explain narrative incoherence, as well as symptoms such as emotional dysregulation and cognitive biases. Conclusions: The difficulty for BPD subjects in producing a coherent personal narrative has been interpreted as a correlation of anomalies in autobiographical memories and consequently identities, which were equally compromised by the experience of discontinuity in the temporal structure. This would confirm the hypothesis of the temporal fragmentation of the self in BPD. Although some limitations have been encountered, we suggest that the understanding of identity diffusion and lived time in BPD subjects could represent a useful guide for further research.

## 1. Introduction

Borderline Personality Disorder (BPD) is a complex, heterogeneous psychopathological condition that significantly affects people’s lives as it impacts both intrapersonal and interpersonal areas of their existence. BPD is characterized by affective instability, difficulty in managing relationships and daily commitments, and identity disturbances [1]. Its prevalence is estimated to be between 0.7% and 2.7% [2], with approximately 20% of diagnosed individuals being psychiatric inpatients [1], with evident consequences in the healthcare burden.

The literature acknowledges the multifactorial nature of this disorder, as well as its phenotypical heterogeneity. Even if different interpretative models have been proposed, there is agreement in considering identity disturbances, such as identity diffusion, fragmentation, and instability, as core features of BPD. In particular, we explored the model of BPD as a disorder of narrative identity and temporal fragmentation. For this purpose, we performed a systematic review following PRISMA 2020 guidelines. Our objective was to synthesize the current literature about the relationship between identity disturbances and lived time in adult patients with BPD.

### 1.1. Borderline Personality Disorder as a Disorder of Narrative Identity

Psychodynamic research has described identity diffusion, meant as a difficulty in producing a coherent and temporally continuous experience of oneself, as one of the main features of BPD. Although this phenomenon is also present in other personality disorders, it takes on specific modalities in persons with BPD. According to Wilkinson-Ryan, experiences of painful inconsistency, role absorption, objective inconsistency, and lack of commitment may be considered specific of BPD [3]. These elements indicate the difficulty for patients to perceive a continuous and coherent self within their life history, leading them to assume social masks to meet others’ expectations, or to engage in self-harming in a desperate attempt to feel, or to continuously change their mind and life plans.

From a phenomenological point of view, BPD had been described as a disorder characterized by “Temporal Fragmentation” [4], which means that BPD subjects would experience a fragmentation in their temporal continuity, impacting the development of their narrative identity. Narrative identity is considered a particular kind of identity, which, according to the philosopher Ricoeur [5], is accessible through narrative function. Everyone produces a narrative of oneself, including his or her personal history, life events, and the personal representation of who he or she is. The narrative function is mediated by the ‘active’ temporal synthesis described by Husserl [6]: while the ‘passive’, pre-reflexive and involuntary temporal synthesis serves to perceive objects as integrated, which means to connect their details, perceived in succession, in a whole perception, through the ‘active’ synthesis, which is conscious and voluntary, I establish more or less coherent formulations about who I am, who I was, and who I will be.

According to Fuchs’ model, BPD patients would enact temporal self-splitting as a defensive mechanism, excluding the past and the future as dimensions of commitment, consistency, and responsibility, and thus experiencing identity fragmentation. In particular, fragmented self-narration would be able to explain different clinical features, such as affect dysregulation, impulsivity, instability in relationships, and the chronic sense of emptiness [4].

The capacity to produce a narrative about oneself is built on autobiographical memory, which develops from childhood within social and cultural contexts [7]. As a result of this process, ‘narrative identity’ ends up being the result of the continuous and endless reflective work of narrating one’s own story and arranging one’s experiences and actions in a meaningful order. Autobiographical memory allows for the interpretation of past events to create a personal story that takes into account connections between past, present and future, thus serving as an index of identity integration [8]. It is possible to hypothesize that more fragmented identities are related to more disintegrated memories, and that the process of ‘active’ temporal synthesis—the integration between past, present, and future perspectives—is altered in BPD patients.

Following the hypothesis that narrating one’s life story allows people to obtain a coherent narrative identity over time [9], it is not surprising to find the narratives of BPD subjects to be incoherent, unable to meet their emotional needs and characterized by more negative content [10,11]. In particular, a correlation between identity diffusion and deficits in autobiographical memory detectable from disorganized, incongruent, and approximate narratives has been described [9,12,13,14,15]. Fragmented narrative identity fails to be related to a healthy memory system, characterized by specificity, integration, positive contents, and dependent on the ability to verbalize experiences [16]. Generally, more coherent autobiographical memories are associated with positive effects on psychological well-being [7,17,18,19].

In a longitudinal study by Lodi-Smith and colleagues [14], personality traits were found to influence how people form narratives about themselves, confirming that maladaptive traits are related to alterations in life-story narrations. Moreover, some authors like Blagov and Singer [20] have reported a negative association between memory specificity and repressive defense mechanisms, leaving open the hypothesis that a less detailed narration of painful memories would protect the subject from the anguish they evoke, while high specificity associated with high–moderate levels of self-control would indicate good cognitive abilities.

Nevertheless, narrative coherence is also connected to good mentalizing ability, considered the metacognitive and affective process that, through reflective functions, allows for the identification of one’s own and others’ mental states, reasoning about them, and coherently interpreting reality [13,21]. According to this perspective, we consider the aspects of metacognition strictly associated with mentalization processes, such as the ability to understand ours and others’ thoughts and intentions and thus to develop a coherent representation of one’s identity [13]. Fonagy [22] argues that negative early experiences (i.e., maltreatment, neglect, lack of attunement with primary caregivers) compromise the reflective capacities of the child, impacting the ability to evaluate self and other representations in an attachment context, and to make them understandable through language [23]. Deficits in mentalization would manifest not only in the difficulty to empathize and understand others’ perspective, but also in the difficulty of self-understanding [23]. The development of a stable and coherent sense of identity is thus negatively impacted, resulting in a disruption of the narrative ability to produce autobiographical stories [5,7]. In patients with BPD, early adverse events are frequently described [24,25,26] and are supposed to play a significant role in the etiopathogenesis of the disorder [27]. Also, BPD patients’ difficulty to adequately integrate autobiographical memories and coherently narrate them might be compromised by their inability to enter others’ mind and to understand their intentions [28]. The consequence would be the known tendency of anticipating or interpreting others’ intention according not to reality but to their own negative expectations [29].

### 1.2. Emotions and Lived Time of Persons with BPD

In such a context, unfolding the lived experience of persons with BPD becomes a fundamental tool for understanding and treating their condition [30,31,32,33].

Specifically, the way patients with BPD experience themselves and the world—that is, their “life-world” [34,35]—is first and foremost characterized by two basic emotions—a permanent trait of dysphoric mood occasionally interrupted by angry outbursts [32,36,37]. While anger is a defined emotion, which generally responds to a personal offence, dysphoria is characterized by more subdued manifestations and a less direct connection to behavioral patterns [38]. Dysphoria can be succinctly described as a state of ‘chaotic lethargy’ [39], an oxymoronic image which encapsulates the dysphoric persons’ inability to pursue their goals, passive expectation and clouded self-awareness. The term ‘lethargy’ should not be equated with a sense of emotional indifference; rather, the dysphoric person is overwhelmed by over-intense feelings and the despairing sense of a time waste.

Depending on the current emotional tone, Stanghellini and Mancini [40] have distinguished two basic configurations of the life-world of persons with BPD. Lived space ranges from being “isotropic” when dysphoria prevails to being completely occupied by the offender when anger comes to the forefront. Bodily experiences associated with dysphoria include feelings of self- and body fragmentation and attempts to compensate for this through aggressive and self-injurious acts [40,41,42]. When patients with BPD are dysphoric, a view of the self as a victim prevails or it lacks cohesion [43], whereas the other person may be experienced as inauthentic or as a humiliating executioner [44,45].

However, considering the purpose of the present study, our aim is to focus on lived time; that is, how each individual experiences temporality. When dysphoric mood prevails, lived time can be characterized by monotonousness and instantaneousness. In the former case, temporal perspectives cannot be clearly distinguished, resulting in a sense of profound boredom; in the latter case, time becomes an absolute “now” that hinders the construction of a narrative identity, as the patient’s story collapses into it: their experiences become transient, immediate, and dominated by impulses of the moment [40,46,47]. Kimura [48] uses the term “intra festum” to signal the eruption of ecstasy—as in a festive atmosphere—emerging abruptly from a stagnant present time.

According to Fuchs [4], the total identification with the present moment leads BPD patients to fragmentation of the narrative self, followed by a fluctuating view of the self with rapidly changing roles and a sense of emptiness, hindering consideration of the effects of their actions and leading to guiding reference patterns. When the state of anger prevails, time changes and becomes an absolute urgency for vengeance without any concern for the consequences of one’s acts [40]. According to Lo Monte and Englebert [49,50], the failure to articulate temporal planes exclusively in favor of the current one is attributed to the inefficiency of cognitive functions in BPD patients, particularly in thinking and language, although this allows them to distance themselves from unpleasant feelings.

### 1.3. Research Question

The aim of the present review is to synthesize the current literature about the correlation between lived time and identity disturbances in adult patients with BPD. The research question was as follows: “What are the known relationships between the altered experience of lived time and identity disorder in subjects diagnosed with BPD?”.

## 2. Material and Methods

### Selection Procedure and Inclusion Criteria

The present study was designed as a systematic review, aimed at synthesizing available research on the experience of temporality in patients with BPD. This systematic review was performed following PRISMA 2020 guidelines. Three scientific databases—Pubmed, Science Direct and PsycInfo—were consulted using the following keyword strings and Boolean operators: “Borderline Personality Disorder AND lived time”, (Borderline Personality Disorder AND narrative identity) NOT adolescence, Borderline Personality Disorder AND identity AND phenomenology, Borderline Personality Disorder AND autobiographical memories, Borderline Personality Disorder AND metacognition.

For this search, the following inclusion criteria were used: (1) adult subjects (age over 18 years), (2) English language, (3) published between 2013 and 2023, (4) empirical research, (5) open access, and (6) focusing on BPD without comorbidity with other psychiatric disorders.

The exclusion criteria were as follows: (1) subjects under the age of 18, (2) articles not in English language, (3) articles published more than 10 years ago or in press, (4) study design (reviews and meta-analyses), (5) comorbidity with other psychiatric disorders, (6) articles not open access, (7) disorders other than BPD, and (8) studies focused on BPD treatment.

In particular, the following filters were used: on PubMed, 10 years (2013–2023), English, free full text, human; on ScienceDirect, 2013–2023, English, open access and open archive, Psychology; and on PsycInfo, 2013–2023, English, open access. Only those articles dealing with both temporality and identity were included in the present review. In this paper, we fully addressed the issue of the integration of neurological and psychosocial models, which is certainly extremely relevant but falls outside the focus of our research whose main scope is to discuss the interrelations between temporality and narrative identity in BDP people.

For the article selection procedure, the PRISMA guidelines (Moher et al., 2015) were followed, summarizing them graphically through a flowchart.

The selection procedure was conducted by IF and CME, under the supervision of GS.

Consulting the three databases, 2176 articles were identified, of which 167 were on Pubmed, 1844 on Science Direct, and 165 on PsycInfo. No additional articles were added through other sources. After applying the inclusion criteria, we obtained 142 articles, including 27 from Pubmed, 83 from Science Direct, and 32 from PsycInfo, which were analyzed by title and abstract; of these, 109 were excluded. Subsequently, 33 full-text articles were evaluated, including 16 from Pubmed, 3 from Science Direct, and 14 from PsycInfo, and 18 were excluded for not meeting some of the inclusion criteria. Therefore, the articles included were 15, including 11 from Pubmed, 0 from Science Direct, and 4 from PsycInfo.

**Figure 1:**
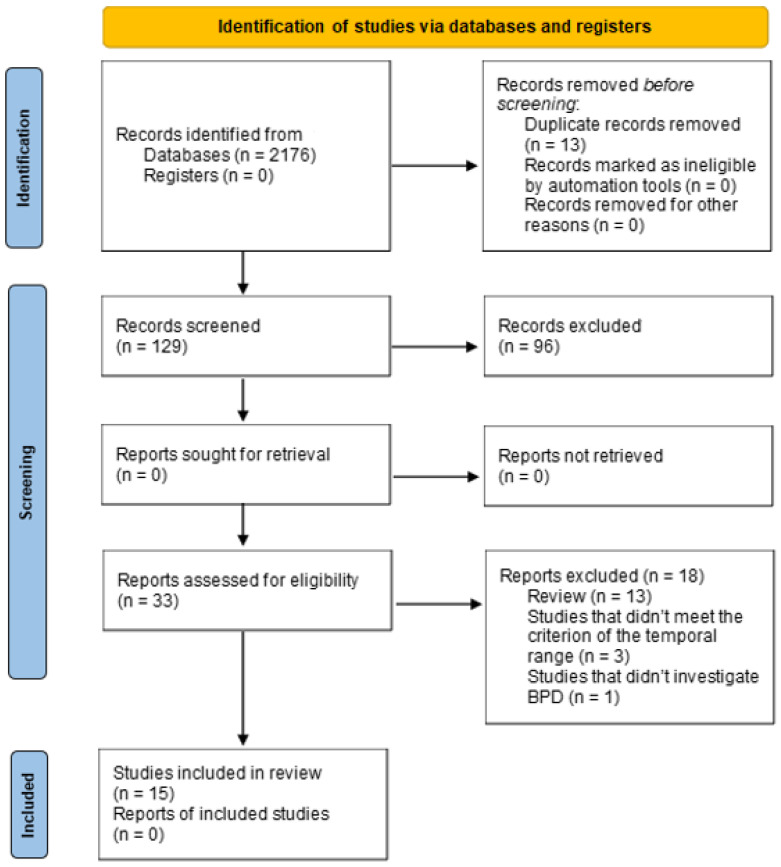
PRISMA 2020 flowchart of the search strategy.

The selection procedure is represented and further explained in Figure 1.

This research was finalized on 6 March 2024. No automatic software was used for article removal.

## 3. Results

At the end of the selection procedure, 15 articles were included in this review, including the following thematic categories: (Section 3.1) studies investigating the structure and content of autobiographical narratives; (Section 3.2) studies highlighting alterations in temporality; (Section 3.3) studies investigating mentalization; (Section 3.4) studies detecting brain alterations and dealing with identity and temporality.

The results are summarized in Table 1 and Table 2.

### 3.1. Studies Investigating the Structure and Content of Autobiographical Narratives

Guruprasad and Bhola [51] administered biopsychosocial interviews to five patients with BPD at the beginning of psychotherapy to code their narratives, which were found to be relatively uncomplicated and unspecific, especially regarding traumatic or painful memories, with prevalent themes of contamination (events starting positively but ending disastrously), and poorly integrated: causal and temporal connections were fragmented even when recounting events occurring over long periods, showing difficulties in creating networks of meanings. Jørgensen and Bøye [53] focused on the subjective experience of identity diffusion in 16 women, who were asked through semi-structured interviews to describe themselves alone or in the presence of others, the most important elements of their lives, and their future expectations. The obtained descriptions allowed for the identification of different categories of self-representations, refuting the idea that BPD patients would be unable to narrate aspects of their daily life or inner experiences since, in some cases, they even expressed themselves through metaphorical language. However, the ability to respond to questions about their identity was still compromised. This is confirmed by the research of Bendstrup et al. [57], who analyzed the written autobiographical memories of 26 women with BPD and 28 healthy women aged between 18 and 45 years. The authors found them to be less detailed and more disorganized in terms of coherence, which was more evident if they had experienced adversity during childhood.

Other authors analyzed the individual turning point memories of participants—i.e., those experiences that changed their lives or the type of person they are. Vanderveren et al. [55] investigated the association between the coherence of such memories with psychological well-being, identity functioning, and personality disorder symptoms. Both patients with BPD and patients with antisocial personality disorder (ASPD) were considered in a sample of 333 adults aged 18 to 30 years. According to this study, narrative coherence does not significantly contribute to predicting BPD symptoms; so, the incongruity present in the stories could be a distinctive feature of disturbed identity functioning in general, and particularly of patients with ASPD. Demonstrating the contradictory outcomes reported by studies in the literature, Sajjadi et al. [55] obtained the opposite result by examining, through self-report measures, the relationship between BPD traits and various manifestations of disturbed identity in a non-clinical sample of 298 university students. Participants with BPD traits (such as hostility and anxiety) were found to produce narratives with a lower degree of agency, while narratives were more fragmented in the presence of intrapersonal and interpersonal difficulties, in terms of identity and intimacy.

Analyzing the content aspect of autobiographical narratives, it is possible to consider trust as a fundamental element in the lives of BPD patients. Botsford and Renneberg [28] examined the narratives of 36 patients with BPD and 99 healthy controls; subjects with BPD, in addition to presenting memories with mainly negative or conflicting emotions, mostly recalled events where their trust was betrayed by family members or partners. In particular, they found that patients with BPD reported a greater number of events where they could not trust a person’s emotional reliability, which includes empathic components and understanding of others, supporting the presence of deficits in mentalization. Conversely, focusing on the analysis of autobiographical memories of rejection in a sample composed of 30 patients with BPD, 27 patients with Major Depressive Disorder, and 30 healthy controls, Rosenbach and Renneberg [58] highlighted a higher level of sensitivity to rejection in subjects with BPD, resulting in less specific, shorter, and more self-referential memories.

### 3.2. Studies Highlighting Alterations in Temporality

Sterna and Moskalewicz [39] investigated the process of mental adaptation to the diagnosis through semi-structured phenomenological interviews and the Cottle’s “Circles Test” on a sample of 10 women diagnosed with BPD aged between 20 and 32 years. They observed the discontinuity of the underlying temporal process: past, present and future appeared separate, marked by emotional peaks of different intensities. The recollection of the past was characterized by profound feelings of pain, anger, and guilt and by the almost total absence of agency—which the authors termed “chaotic lethargy”. The present, when the patients were informed about their diagnosis, was characterized by an increased sense of agency—described as “awakening from chaotic lethargy”—gradually raising the patient’s awareness of their symptomatic condition and reducing the intensity of emotional peaks. The future, on the other hand, began with the end of treatment and remained indefinite due to the possibility of developing according to two intrapsychic scenarios: one in which the patient regressed to the previous symptomatic condition and one opposed, where, instead, they managed their disorder better. The interesting aspect of this study is the possibility of modifying the relationship between the self and symptoms through the communication of the diagnosis, which allows for their recognition in order to exclude from the healthy part of the patient. These alterations in temporality coincide with the phases of adaptation to the diagnosis: in the past, feelings of inadequacy and anguish prevailed, and in the present, there were intense reactions at the time of its communication, while in the future, there was self-fragmentation, as the ambivalent feelings of the patient experienced regarding the diagnosis (e.g., rejection versus identification) conflicted.

Temporal discontinuity is also reported by Bendstrup et al. [57], who observed the absence of temporal structure as events were causally and temporally disconnected.

### 3.3. Studies Investigating Mentalization

Saldanha-Silva et al. [61] investigated the relationship between maladaptive beliefs, personality traits, and symptoms of BPD by administering specific questionnaires to a non-clinical sample of 823 adults aged between 19 and 39 years. The results suggest that specific personality traits such as high neuroticism and low conscientiousness are associated with the frequency of BPD symptoms and predict them; moreover, symptoms are mediated by maladaptive beliefs. The use of questionnaires allowed for the investigation of the relationship between insight traits, metacognition, and impulsivity, as carried out by Martin and Del-Monte [62] on a sample of 190 patients with BPD (180 females and 10 males) with a mean age of 35 years. In addition to confirming impulsivity in borderline patients, the authors found these patients’ metacognitive abilities to be functional, with higher values compared to those obtained by healthy controls or other clinical groups present in the literature. The level of insight in patients with BPD was equivalent to that of patients with other disorders. The results in the current scientific literature are contradictory: Cyrkot et al. [63] highlighted the dysfunction of metacognitive processes in persons with BPD by investigating them through the Reading the Mind in the Eyes Test (RMET) (33 BPD patients and 33 healthy participants), assessing the ability to recognize others’ mental states based on information gathered from their facial expressions. The study revealed that patients with BPD obtained lower scores on the RMET task, resulting in less accurate recognition of proposed mental states, while confidence in their metacognitive judgments measured through post-decision betting did not differ from the non-clinical group, suggesting similar loss aversion strategies. Despite this, people with BPD showed higher levels of accuracy when recognizing positive elements that were not, thus being more confident in their decisions even when they were wrong, failing to modify them and thus confirming dysfunctional metacognitive functioning.

Another study was included in our review that deviates from these as it did not involve a sample of patients strictly diagnosed with BPD. Van Schie and colleagues [60], hypothesizing that greater vividness correlated with mood improvement and that the effect was stronger in the case of positive memories, asked 47 non-clinical subjects of various ages to write four positive and four neutral autobiographical memories in the first person and at the present time in order to also investigate the role of autonoetic consciousness by allowing participants to relive them later through an episodic memory task. From the analyses conducted, it emerged that greater vividness correlated with longer, more pleasant, and more distant memories, mediating a more immersive mode of reliving them; furthermore, mood improved after reliving positive memories, as did state self-esteem. Despite not focusing on BPD, this study is important for having highlighted the positive impact of reliving vivid memories on mental well-being, a clinically relevant aspect even with patients with BPD because it would allow these to work on their memories to make them more integrated and detailed, thus resulting in protective factors in moments of crisis, improving mood, and facilitating the process of temporal synthesis.

### 3.4. Studies Detecting Brain Alterations and Dealing with Identity and Temporality

Some researchers have sought to provide a neurobiological basis for narrative incoherence or hypergeneralization of memories using neuroimaging techniques. Bozzatello et al. [52] examined the difference in brain activity in 23 patients diagnosed with BPD who had obtained a score indicating highly disturbed identity on a questionnaire assessing its integrity, and 22 healthy controls through functional magnetic resonance imaging (fMRI) sessions during the presentation of two resolved life events, i.e., events that the subjects had processed and integrated into their experience, and two unresolved life events that they had not integrated. Functional connectivity alterations were found in both experimental conditions, particularly involving the anterior cingulate cortex (ACC) and dorsolateral prefrontal cortex (DLPFC)—areas involved in autobiographical narrative formation processes, as they allow, in addition to the integration of affective and cognitive elements, for the retrieval of memories.

These results are complemented by those supporting pre-existing brain alterations: Soloff et al. [56], after selecting a sample of 23 individuals with BPD and 15 healthy controls aged between 18 and 45 years, explored the neural bases of the mechanism of interference of negative affectivity on cognitive processes, corresponding to a greater functional impairment of areas involved in this apparatus. They used fMRI sessions with the addition of emotive stimuli and an episodic memory task, demonstrating that in these patients, negative affectivity selectively altered the response of brain regions, impacting executive processes in terms of reduced focused attention, decision-making mechanism, response inhibition and episodic memory processes.

Similarly, Szczepaniak et al. [59] investigated the role of negative affective stimuli, considering their effects on neural priming, knowing that familiar stimuli induce greater priming. The study sample included 40 subjects with BPD and 25 healthy controls aged between 18 and 44 years, undergoing fMRI scans while performing an episodic memory task involving encoding and subsequent recognition of presented inputs. Patients with BPD showed greater priming for negative-valence stimuli, demonstrating alterations in the recognition mechanism of affective stimuli.

## 4. Discussion

All articles analyzed in this review have highlighted, on the one hand, the close connection between identity disturbances, and, on the other hand, both the ability to produce coherent autobiographical narratives and the lived experience of time. When presenting a psychopathological condition such as BPD, significant alterations in the lived experience of time and in the narrative identity have been described [28,39,51,52,53,54,55,56,57,58]. The lack of integration of memories and therefore of narratives would lead to considering the main core of BPD a disorder of the temporal synthesis, which implies the incoherent integration of past, present, and future [39]. In fact, our identity construction is based on the ability to produce a synthesis between what we have been (*retentio*), what we are (*praesentatio*), and what we expect to be (*protentio*) [64]. This hypothesis is confirmed by studies investigating the structure and content of autobiographical narratives [51,53,54,55,57,58], which revealed features of moderate complexity, specificity, and integration—particularly regarding self-representation and interpersonal relationships. The difficulty of producing a temporal synthesis of past–present–future and therefore a coherent and integrated representation of one’s identity has been correlated to various factors, both biological, psychological and social [25]. The most strongly demonstrated correlation, as we will see, is that with early trauma, which, by abruptly breaking in, disturbs the temporal and identity organization in the process of being formed [24]. Different theoretical perspectives, among which that of Meares [65] is of particular relevance, state that traumas cause dissociative discontinuities in the child, which impact the narrative structuring of identity in the long term.

Furthermore, mainly negative contents emerged from the thematic analysis. These included both memories defined as contamination [51] and actual experiences of abuse [57], rejection or anger [58], or situations where the trust of persons with BPD was betrayed by close people such as family members or partners [28]. In particular, Bendstrup et al. [57] associated reduced narrative coherence—also stemming from inadequate use of temporal and causal connections—with early trauma because participants provided summary information about the context that the reader needed to know for adequate understanding of the story told. Since they provided approximate descriptions on the events that had occurred previously, on the main characters, and on the space and time of their development, the authors hypothesized that disturbing memories—including traumatic ones—being emotionally intolerable, might be poorly integrated and thus described with less coherence [51], supporting the notion of the protective function of narrative incoherence [20]. There is evidence in the literature that abuse compromises mental health, reflective capacity, and the self-esteem of the child [22,29], increasing the likelihood over time of developing BPD and therefore suffering from identity diffusion. However, this aspect deserves further investigation because, at the same time, Vanderveren’s research group [54] found no association between narrative coherence and BPD, which, instead, was described in subjects with ASPD. This finding leaves open the hypothesis that thematic, chronological, contextual, and causal incoherence may not be specific to BPD but related to all psychopathological conditions where identity disturbances are central. Contrary to what has just been outlined, Sajjadi et al. [55] found narrative identity to be the main predictor of BPD characteristics: indeed, being characterized by identity diffusion, patients produced stories with a lower degree of agency and realization of agency; so, the protagonists did not take an active role in changing events or satisfying their own needs.

Supporting this idea, it has been found that, despite the narratives of BPD patients demonstrating their ability to produce understandable and relevant stories about their lived experiences, they still contain contradictory self-representations as a result of identity disturbances [53]. The thematic categories mainly identified concerned the self as a disintegrated core, mutable, excessively compliant to others’ expectations due to constant fear of rejection, vulnerable, defective, lacking agency and guiding values. These themes are accompanied by intense feelings of emptiness, loneliness, and lack of the necessary closeness of contact with others as confirmation of their own existence, although these patients are often unable to manage the relationships sought even through a dysfunctional way of experiencing intimacy, both emotional and sexual. 

It is important to consider the current knowledge regarding the relationship between early adverse experiences with attachment figures and the consequent difficulty in constructing a coherent narrative identity following developed mentalization deficits [22,29] which would result in incoherent narratives. Therefore, it is thought to be important in the therapeutic process to analyze patients’ life events in general and especially in those with BPD, as it would increase the feeling of being listened to and understood by the clinician [51]. Less complex stories often lacked emotional content; therefore, it would also be useful to investigate a possible association between the ability to remember the details of an event—which increases its complexity—and the emotions associated with it to understand if the type of emotional valence could invalidate the memorization process, making it more fragmented [66].

Other studies, although not focusing on narrative coherence or disturbed identity [39,52], indirectly provided important information on the narratives produced by persons with BPD. For example, Sterna and Moskalewicz’s qualitative study [39] allowed for a better understanding of the emotional world of patients before, during, and after receiving their diagnosis.

Their results should be taken into account for treatment strategies: given the difficulty of patients in perceiving themselves cohesively over time, recognizing the need to start a treatment journey could be a first anchor in the present moment, able to increase awareness of their disorder. By doing so, they could temporally distinguish what happened before the diagnosis, classifying it as past, from what will happen afterward, classifying it as future, and with the help of professionals, manage any intrusive memories or unpleasant emotions that may arise. Marking the future time as the end of the treatment would also help considering long-term projects.

The neurobiological perspective of borderline functioning presented by Bozzatello et al. [52] has also provided support for the hypothesis that narrative modes with a lower degree of coherence of past life events are related to their symptomatology. Alterations in brain activity in the ACC and DLPFC, both hyperactivated (with the addition of two other areas of fundamental importance such as the HIP for memory processes and the OFC for cognitive control), were also found by Soloff et al. [56], allowing inferences to be drawn about the abilities that patients with BPD have in recalling events compared to non-clinical controls. Since negative emotional context had the greatest effect on cognitive processing, this would confirm emotional dysregulation with consequent cognitive biases underlying the preferential selection of negative stimuli in people with BPD. Consistent with this, Szczepaniak et al. [59] showed the greater familiarity that subjects with BPD have with negative stimuli: when exposed to negative-valence images, they showed greater neural priming.

In line with the objective of this review to analyze the disturbed identity of BPD patients through autobiographical memory, two characteristic aspects of these individuals that inevitably impact the process of encoding, storing, and recalling memories have been considered: affective instability and metacognitive deficits. For this reason, studies supporting the existence of so-called emotional dysregulation in patients with BPD have been reported, which could be at the basis of their biases toward negative emotions or stimuli [56,58,59], or of a greater frequency in recalling experiences where they felt betrayed [58], as well as studies demonstrating the presence of metacognitive deficits and habitual dysfunctional beliefs [61,62,63].

A significant relationship has emerged between insight and metacognition on BPD traits—and, especially, impulsivity. The outcomes of the two studies reported on the metacognitive abilities of borderline patients have resulted in contrasting views: while Martin and Del-Monte [62] found functional metacognitive abilities in subjects with BPD compared to healthy controls and a level of insight comparable to theirs, Cyrkot and colleagues [63] highlighted dysfunctional patterns that seem to be involved in theory of mind mechanisms and metacognitive ones. In particular, the processes of understanding and attributing mental states would be deficient, as well as those of controlling and monitoring information following overestimation of one’s confidence when giving incorrect answers: such alterations would ultimately influence behavior, confirming hypotheses regarding cognitive anomalies and the difficult management of impulses in BPD patients.

However, if the results of the Cyrkot group [63] were further confirmed in the future, clinicians could help patients with BPD become aware of their internal states to understand that the difficulties experienced are mainly caused by their evaluations rather than external reality [66], thus making symptomatic improvement possible through cognitive restructuring processes of their narratives (e.g., victim–perpetrator).

Finally, the study by van Schie and colleagues [60], although it did not select a sample of individuals with BPD, provided useful data to advance treatment proposals. If future studies would confirm that the act of reliving positive memories determines an improvement in mood as a result of increased vividness of memories and increased autonoetic consciousness, these latter two aspects could also positively impact other characteristics of borderline symptomatology, for example, process of construction of autobiographical narratives, the awareness of lived time, and the management of difficult social relationships. The results obtained suggest that there are different factors that influence the experience of reliving personal memories, each with an independent effect from the other: vividness, length, pleasantness, and remoteness. However, since greater vividness correlated with longer, more pleasant, and more distant memories, it might be important to investigate whether these characteristics are also present in patients with BPD to highlight them during treatment or try to bring them out where they are latent. Since the main symptoms of BPD include a disturbed sense of self, unstable social relationships, and affective and behavioral dysregulation [1], it is likely that the functional properties of autobiographical memories are compromised in these patients. With low levels of trait self-esteem being associated with less specific memories [60], the fragmentation of those of patients with BPD could be due to poor confidence in their abilities and low self-esteem rather than to identity diffusion or metacognitive deficits. However, this hypothesis should be further empirically tested. Finally, since the most vivid memories and better mood activated brain regions such as the amygdala and hippocampus, which are structurally anomalous in subjects with BPD [56,67], in them, memories with the mentioned attributes could activate other areas, or the same ones but in a not significant way.

## 5. Limitations

Various limitations were identified in each study, inevitably impacting the validity of this review. Methodological limitations encountered during selection (such as the type of criteria chosen and the number of databases) include those related to sample characteristics deemed unrepresentative due to small size, inadequate representation of the gender ratio, homogeneity, absence of patients diagnosed with BPD, or comorbidity with other disorders. Our choice to include only articles dealing with temporality and narrative identity led to the exclusion of relevant articles on metacognition, which have already found a more complete review elsewhere [62,68]. There are also limitations regarding the psychometric validity of the instruments used or the choice to use only qualitative or quantitative ones, research design, lack of a clear and unambiguous definition of the constructs examined, failure to assess the effect of medications taken by some patients, and absence, in some cases, of a control group.

## 6. Conclusions

The theoretical rationale underlying this review is based on the well-known negative impact that BPD has on patients’ daily lives [1], the frequency with which it has manifested in recent years [2,46,49], its interpretation in terms of narrative identity [5] and particularly in terms of temporal fragmentation of the self [4]. The influence of the processes contributing to the formation of a coherent narrative identity on mental health and abnormal mental conditions has been established [4,7,9,10,69], whose fundamental prerequisite consists of the temporal synthesis of past, present and future, and the production of integrated autobiographical narratives. Especially active synthesis seems to be involved—the ongoing process during which one establishes more or less coherent formulations about oneself.

Despite the presence of conflicting results in the literature, a general agreement emerged from the articles examined regarding the close connection between the typical difficulties of patients with BPD both from a phenomenological and cognitive perspective [52,53,55,56,59,62,63,69] and their way of experiencing time, confirmed also by research investigating their narratives that highlighted their discontinuity [39], and certain characteristics such as specificity, complexity, thematic content [28,51,58], or coherence [54,57]. Beyond methodological differences and the different objectives of each study, there was also agreement on the association between disturbed identity and poor narrative coherence, the presence of negative contents, deficits in cognitive and metacognitive areas, and pre-existing metabolic, structural, and functional alterations hypothesized as the neurobiological basis of narrative incoherence. The hypothesis of temporal fragmentation of the self in individuals with BPD [4] was endorsed [39,57].

We can therefore conclude that the objective of this review has been achieved in providing a reassessment of the most updated data on the theme of identity and temporal fragmentation in adult patients with BPD, which can ultimately be considered a disorder of temporal synthesis.

However, since different limitations have been identified, it is advisable that these results be interpreted with caution. We also hope that, in the future, they will be replicated to provide greater validity to the examined studies, but also that further research will be conducted to improve the current theoretical knowledge and obtain solid empirical evidence on which to base future interventions.

## Figures and Tables

**Table 1:** Characteristics of the reviewed studies.

Study	Study Design	Quality	Year	Objectives	Group (Sample Size; Number of Women and Men with Age Range or Mean)	Methods	Main Results
Sterna A. and Moskalewicz M. [39]	Cross-sectional Study	2	2022	Phenomenological descriptionof the adaptation process to the diagnosis in borderline patients, and study of the underlying temporal structure	10 women with BPD. 20–32 years	Phenomenological semi-structured interviews (Giorgi method) + Cottle Circles Test	The adaptation process to the diagnosis involves three phases and simultaneously allows for identifying the patients’ difficulties as symptoms. The temporal structure is discontinuous.
Guruprasad D. and Bhola P. [51]	Longitudinal Study	1	2014	To examinate the structure, content, and quality of narratives concerning autobiographical memory	5 patients with BPD: 4 women and 1 man. 23–44 years	Bio-psycho-social interviews during the initial phase of their therapy	The structure of the narratives was found to be moderately complex, somewhat unspecific, predominantly autobiographical, and poorly integrated, with dominant themes of contamination.
Bozzatello P. et al. [52]	Cross-sectional Study	2	2019	To investigate differences in brain activity between borderline patients with identity diffusion and healthy controls	24 patients with BPD: 15 women (62.5%) and 9 men. Mean_age_ ± SD = 37.17 ± 13.23 24 healthy controls: 13 women (54.16%) and 11 men. Mean_age_ ± SD = 36.36 ± 12.85 18–60 years	fMRI analysis during the presentation of resolved and unresolved life events + Witzel autobiographical interview (1985)	Increased brain activity in both conditions across various areas in subjects with BPD. Hyperactivation of the ACC and DLPFC, perhaps indicating an inability to reconstruct coherent narratives.
Jørgensen C. R. and Bøye R. [53]	Cross-sectional Study	2	2022	To investigate the subjective experience in patients with BPD of identity diffusion, as a central aspect of their pathology	16 women with BPD. Mean_age_ ± SD = 27.6 ± 6.2 21–43 years	Semi-structured interviews analyzed using phenomenological method	The manifestation of identity diffusion has been classified into nine categories, based on the management of various aspects of the self and relationships with others.
Vanderveren E. et al. [54]	Cross-sectional Study	3	2021	To analyze the associations between narrative coherence (NC) and psychological well-being, identity functioning, and personality disorder (PD) symptoms. To investigate whether such coherence predicts unique variance in PD symptoms beyond identity and interpersonal functioning	333 adults: 93 men (27.9%) and 240 women (72.1%). Mean_age_ ± SD = 22.56 ± 3.13 18–30 years	Online survey for the analysis of turning point recall + specific self-report questionnaires to assess psychological well-being, identity functioning, and symptoms of PD; specifically focusing on BPD, ASPD, and interpersonal functioning	The narrative incoherence in memories could be a characteristic of disturbed identity functioning.
Sajjadi F. S. et al. [55]	Cross-sectional Study	2	2022	To examine the relationship between features of BPD and the various manifestations of disturbed identity, considered as narrative identity	298 University student without personality disorders: 211 women Mean_age_ ± SD = 21.22 ± 3.12 and 87 men Mean_age_ ± SD = 21.55 ± 3.80 21–22 years	Self-report measures for BPD characteristics + Turning-point interview for narratives	The narrative identity emerged as the dominant predictor of BPD characteristics.
Soloff P. H. et al. [56]	Cross-sectional Study	2	2015	To examine the neural bases of the mechanism of interference of negative affectivity on cognitive processes	23 women with BPD. 15 healthy controls. 18–45 years	fMRI scans during the protocols Affective Go No-Go, X-CPT, and an affective episodic memory task	In borderline individuals has been observed hyperactivation in areas relevant to Go No-Go and X-CPT tasks, and hypoactivation in those related to memory. These functional deficits are linked to structural or metabolic abnormalities.
Botsford J. and Renneberg B. [28]	Cross-sectional Study	2	2020	To investigate trust experiences in patients with BPD	36 patients with BPD: 30 women and 6 men. Mean_age_ ± SD = 29.6 ± 9.9 99 healthy controls: 70 women and 29 men. Mean_age_ ± SD = 39.1 ± 17.9	Self-report measures for thematic analysis of autobiographical memories of trust and well-being index (OSM-5)	Subjects with BPD mainly recall events where their trust is betrayed by family members or partners. The theme of trust in their memories is crucial for them.
Bendstrup G. et al. [57]	Cross-sectional Study	2	2021	To investigate whether the memories of subjects with BPD have lower narrative coherence, and whether this is associated with childhood trauma	26 women with BPD. Mean_age_ ± SD = 28.67 ± 7.21 28 healthy controls women. Mean_age_ ± SD = 28.88 ± 8.77 18–45 years	Analysis of written autobiographical memories + questionnaires for assessing childhood trauma + verbal comprehension index	The memories of patients with BPD had reduced narrative coherence (inadequate orientation towards narration and lack of a narrative structure). This was associated with childhood traumas.
Rosenbach C. and Renneberg B. [58]	Cross-sectional Study	3	2014	To identify the difference in the quality of rejection memories between patients with BPD and Major Depressive Disorder (MDD), in terms of specificity and language	30 patients with BPD: 28 women and 2 men. Mean_age_ ± SD = 30.5 ± 8.43 27 patients with con MDD: 18 women and 9 men. Mean_age_ ± SD = 41.6 ± 14.5 30 nonclinical controls: 22 women and 8 men. Mean_age_ ± SD = 33.0 ± 10.4	Rejection Sensitivity Questionnaire (RSQ) + Autobiographical Memory Test (AMT) + Linguistic Analysis Software (LIWC)	Patients with BPD recalled less specific, self-referential memories, mainly concerning social environments and featuring frequent words of anger, assessing rejection episodes as more relevant.
Szczepaniak M. et al. [59]	Cross-sectional Study	3	2021	To investigate the effects of the affective valence of stimuli on neural priming, and whether these distinguish patients with BPD from healthy controls	40 patients with BPD: 34 women and 6 men. Mean_age_ ± SD = 31 ± 7.5 25 healthy controls: 23 women and 2 men. Mean_age_ ± SD = 24 ± 6.0 18–44 years	Episodic memory task + fMRI	Subjects with BPD showed greater neural priming for stimuli with negative valence (because they are more familiar to them) and less for those with positive valence.
Van Schie C.C. et al. [60]	Cross-sectional Study	3	2019	To investigate which brain patterns and memory characteristics facilitate the effectiveness of reliving positive autobiographical memories for mood and sense of self	47 women with similar age, education and self-esteem levels. Some of them had mental disorders. Mean_age_ ± SD = 29.36 ± 9.61	fMRI + RAM task (memory), social feedback task, RSES scale (trait self-esteem), MINI-plus (psychopathology)	Reliving positive memories enhances vividness and autonoetic consciousness, which is likely crucial in improving mood.
Saldanha- Silva R. et al. [61]	Cross-sectional Study	2	2019	Analysis of the relationship between maladaptive beliefs, personality traits, and symptoms of BPD, considering beliefs as a mediating factor	823 adults (75% women). Mean_age_ ± SD = 24.09 ± 4.71 18–39 years	Questionnaires for BPD symptoms, beliefs about personality disorders, and socio-demographic profile + Big Five Personality Inventory	The personality traits of neuroticism and conscientious-ness predict BPD symptoms and are mediated by maladaptive beliefs.
Martin S. and Del-Monte J. [62]	Cross-sectional Study	3	2023	Measurement of insight, metacognition, and impulsivity traits in patients with BPD	190 patients with BPD: 10 men and 180 women. Mean_age_ = 35.95	Questionnaires to assess borderline personality (BPQ), metacognitive beliefs (MCQ-30 II), impulsivity (UPPS-S), and insight (BCIS)	A significant relationship has been highlighted between insight and metacognition, both on BPD traits and impulsivity.
Cyrkot T. et al. [63]	Cross-sectional Study	2	2021	To analyze dysfunctional metacognitive processes in patients with BPD	33 patients with BPD: 32 women and 1 man. 33 healthy controls: 31 women and 2 men.	Mind Reading Task (RMET) + scale to evaluate ToM (CR) + scale to evaluate metacognition (PDW)	Individuals with BPD show dysfunctional patterns involving metacognitive processes implicated also in mindreading.

**Note**: Global rating was performed according to these criteria: (1) selection bias (sample size power and number of subjects who agreed to participate into the study); (2) study design (randomized versus non-randomized trials); (3) confounders (Yes/No); (4) blinding (Yes/No); (5) data collection methods (self-reported data, observations by investigators or medical records); (6) presence of description of numbers and reasons for withdrawals and drop-outs. 1 = strong (no weak ratings according to above criteria); 2 = moderate (one weak rating according to above criteria); 3 = weak (two or more weak ratings according to above criteria).

**Table 2:** Main results discussed.

Focus of Investigation	Main Results	Studies
Structure of autobiographical narratives	Association between poor narrative coherence and disturbed identity functioning, fragmented memories, and childhood traumas; Characteristics: - Lower specificity (approximate narratives); - Inadequate narrative orientation (lack of contextual details useful for understanding the event); - Lower complexity (few details, difficulty in representing oneself or interpersonal relationships); - Poor integration (in terms of logical, causal, and temporal connections).	Bendstrup et al. (2021) [57], Guruprasad and Bhola (2014) [51], Jørgensen and Bøye (2022) [53], Rosenbach and Renneberg (2015) [58], Sajjadi et al. (2022) [55], Vanderveren et al. (2021) [54]
Themes of autobiographical narratives	Most frequent theme: contamination; Prevalence of negative contents: mistreatment, rejection, abandonment and betrayed trust, particularly from individuals close to them such as family members or partners; Most reported feelings: anger, fear of abandonment and disappointment.	Bendstrup et al. (2021) [57], Botsford and Renneberg (2020) [28], Guruprasad and Bhola (2014) [51], Rosenbach and Renneberg (2015) [58]
Alterations in temporality (lived time and narrative time)	Information obtained directly by investigating the adaptation process to the diagnosis, or indirectly by analyzing the narratives; Temporal discontinuity: Fuchs’ hypothesis (2007) confirmed.	Bendstrup et al. (2021) [57], Sterna and Moskalewicz (2022) [39]
Functional, structural and metabolic brain alterations in specific regions or some parts thereof	Alterations in functional connectivity underlying narrative incoherence, emotional dysregulation and cognitive biases: - Hyperactivity and hypoactivity in prefrontal areas and in the hippocampus involved in cognitive control and memory processes; - Hyperactivity in the amygdala and ACC implicated in emotional regulation. Some consequences: - Greater familiarity with negative stimuli; - Increased impact of negative affective context on cognitive processing.	Bozzatello et al. (2019) [52], Soloff et al. (2015) [56], Szczepaniak et al. (2021) [59]
Cognitive and metacognitive deficits	Frequent maladaptive beliefs and schemes; Dysfunctional metacognitive processes; Consequences on identity, autobiographical memories and narratives.	Cyrkot et al. (2021) [63], Martin and Del-Monte (2023) [62], Saldanha-Silva et al. (2019) [61]
Role of positive autobiographical memories	Living positive autobiographical memories would increase mood and temporal synthesis; Role of autonoetic consciousness for temporal synthesis.	Van Schie et al. (2019) [60]

## Data Availability

The raw data supporting the conclusions of this article will be made available by the authors on request.
